# Two differentially methylated region networks in nonalcoholic fatty liver disease, viral hepatitis, and hepatocellular carcinoma

**DOI:** 10.1186/s12876-022-02360-4

**Published:** 2022-06-02

**Authors:** Suguru Kurokawa, Masato Yoneda, Yuji Ogawa, Yasushi Honda, Takaomi Kessoku, Kento Imajo, Satoru Saito, Atsushi Nakajima, Kikuko Hotta

**Affiliations:** 1 Laboratory of Pathophysiology and Pharmacotherapeutics, Faculty of Pharmacy, Osaka Ohtani University, 3-11-1 Nishikiori-kita, Tondabayashi, Osaka 584-8540 Japan; 2 Department of Gastroenterology and Hepatology, Yokohama City University Graduate School of Medicine, 3-9 Fukuura, Kanazawa-ku, Yokohama, Kanagawa 236-0004 Japan; 3 Department of Gastroenterology, National Hospital Organization Yokohama Medical Center, 3-60-2 Harajyuku, Totsuka, Yokohama, 245-8675 Japan; 4 Department of Palliative Medicine, Yokohama City University Hospital, 3-9 Fukuura, Kanazawa-ku, Yokohama, 236-0004 Japan; 5 Department of Gastroenterology, Shin-yurigaoka General Hospital, 255 Furusawatsuko, Asao, Kawasaki, 2150-0026 Japan

**Keywords:** Epigenetics, NAFLD, Hepatocellular carcinoma, Network modeling, DNA methylation

## Abstract

**Background:**

We previously reported that two differentially methylated region (DMR) networks identified by DMR and co-methylation analyses are strongly correlated with the fibrosis stages of nonalcoholic fatty liver disease (NAFLD). In the current study, we examined these DMR networks in viral hepatitis and hepatocellular carcinoma (HCC).

**Methods:**

We performed co-methylation analysis of DMRs using a normal dataset (GSE48325), two NAFLD datasets (JGAS000059 and GSE31803), and two HCC datasets (GSE89852 and GSE56588). The dataset GSE60753 was used for validation.

**Results:**

One DMR network was clearly observed in viral hepatitis and two HCC populations. Methylation levels of genes in this network were higher in viral hepatitis and cirrhosis, and lower in HCC. Fatty acid binding protein 1 (*FABP1*), serum/glucocorticoid regulated kinase 2 (*SGK2*), and hepatocyte nuclear factor 4 α (*HNF4A*) were potential hub genes in this network. Increased methylation levels of the *FABP1* gene may be correlated with reduced protection of hepatocytes from oxidative metabolites in NAFLD and viral hepatitis. The decreased methylation levels of *SGK2* may facilitate the growth and proliferation of HCC cells. Decreased methylation levels of *HNF4A* in HCC may be associated with tumorigenesis. The other DMR network was observed in NAFLD, but not in viral hepatitis or HCC. This second network included genes involved in transcriptional regulation, cytoskeleton organization, and cellular proliferation, which are specifically related to fibrosis and/or tumorigenesis in NAFLD.

**Conclusions:**

Our results suggest that one DMR network was associated with fibrosis and tumorigenesis in both NAFLD and viral hepatitis, while the other network was specifically associated with NAFLD progression. Furthermore, *FABP1*, *SGK2*, and *HNF4A* are potential candidate targets for the prevention and treatment of HCC.

**Supplementary Information:**

The online version contains supplementary material available at 10.1186/s12876-022-02360-4.

## Background

Nonalcoholic fatty liver disease (NAFLD) includes a wide spectrum of liver diseases, ranging from the benign non-progression condition non-alcoholic fatty liver to nonalcoholic steatohepatitis (NASH), which can progress to liver cirrhosis and hepatocellular carcinoma (HCC) [1, 2]. Similar to that in other developed countries, NAFLD has become a common disorder associated with metabolic syndrome in Japan [3, 4]. HCC is a significant cause of morbidity and mortality in Japan [5]. Furthermore, HCC is the most common malignancy in patients with NAFLD/NASH, and a higher incidence of HCC has been reported in patients with advanced/severe fibrosis. While the treatment of viral hepatitis improves, NAFLD and NASH are rapidly becoming the leading causes of HCC in Japan. Accordingly, the investigation of NAFLD pathogenesis, especially in relation to HCC, and the discovery of novel treatment targets are of utmost concern.

Recent progress in genetic and epigenetic analyses has provided new insights into NAFLD [6, 7]. Indeed, previous genome-wide association studies by us and others have established a definitive genetic background associated with the development of the disease [8–10]. In addition to genetic effects, changes in epigenetic status are also important for the development of NAFLD [7, 11]. To evaluate the epigenetic status of the NAFLD liver, we previously performed whole hepatic mRNA sequencing followed by weighted gene co-expression network analysis (WGCNA) [12]. We identified two core gene networks involved in NAFLD progression: one contains a scale-free network with four hub genes associated with increases in fibrosis and tumorigenesis and the other consists of a random network associated with mitochondrial dysfunction. One of the most important methods for evaluating the epigenetic status of NAFLD in the liver is DNA methylation analysis. We performed genome-wide hepatic DNA methylation analysis and identified 610 differentially methylated regions (DMRs) that are associated with the progression of NAFLD [13]. We subsequently performed co-methylation analyses of the 610 DMRs and identified two DMR networks associated with NAFLD progression [14]. The annotated genes of one of the networks includes genes involved in transcriptional regulation, cytoskeleton organization, and cellular proliferation and are thus potentially associated with tumorigenesis. The annotated genes of the second network are potentially associated with metabolic dysfunction.

Our previous studies demonstrated that analysis of DMR networks is a powerful tool for understanding the pathogenesis of liver disease and for identifying treatment targets. In the present study, we investigated the methylation levels of fibrosis-associated DMR networks in viral hepatitis and HCC using DNA methylation datasets.

## Methods

### DNA methylation analysis datasets

For DNA methylation analysis, we used the following datasets: our recently published Japanese hepatic DNA methylation data of NAFLD (JGAS000059, http://trace.ddbj.nig.ac.jp/jga/index.html) [13], American hepatic DNA methylation data of NAFLD (NCBI Gene Expression Omnibus accession number GSE31803) [15], Japanese hepatic DNA methylation data of viral hepatitis and HCC (GSE89852) [16], Italian hepatic DNA methylation data of HCC (GSE56588) [17], and German normal hepatic DNA methylation data (GSE48325) [18]. For a validation study of the identified methylated region, we used GSE60753 [19]. Genome-wide DNA methylation levels were determined using the Infinium HumanMethylation450 BeadChip (Illumina, San Diego, CA, USA).

### Co-methylation analysis of DMRs

A total of 610 DMRs, including 3683 CpGs sites, that are differentially methylated between mild fibrosis (F0–F2, fibrosis stages 0 - 2) and advanced fibrosis (F3–F4, fibrosis stages 3 or 4) across the Japanese and American NAFLD cohorts were identified using Probe Lasso [20] as previously reported [13]. Co-methylation analysis was performed as previously described [14] for 610 DMRs of German control (n = 34), Japanese NAFLD (n = 60), American NAFLD (n = 56), Japanese viral hepatitis (n = 37), Japanese HCC (n = 37), and Italian HCC liver (n = 224) samples. Briefly, a co-methylation analysis was performed using WGCNA R package to assess inter-correlation among the 3683 CpG sites [21, 22]. In this analysis, a network was represented by the adjacency matrix *A* = [*α*_*ij*_], where *α*_*i,j*_ was |*PCC*_*i,j*_|^*β*^, *PCC*_*i,j*_ was the Pearson’s correlation coefficient between the methylation levels of CpG probes *i* and *j* across samples, and *β* was a soft threshold. The *β* threshold was set at 16 for the Japanese NAFLD, 14 for the American NAFLD, 7 for the Japanese viral hepatitis, 6 for the Japanese HCC, 6 for the Italian HCC, and 14 for the German control samples, which were the smallest values at which a co-methylation network exhibited scale-free properties with a model-fitting index of *R*^2^ > 0.70. Topological overlap measures were obtained, which were a measure of co-methylation interconnectedness between probes [23]. To evaluate co-methylation interconnectedness between the DMRs, average values of topological overlap measures of the CpG matrix combining two different DMRs were calculated. To extract the DMR networks, the cutoff values of the average values of the topological overlap measures were set to 0.15 for the Japanese NAFLD, American NAFLD, and German control groups, 0.35 for the Japanese viral hepatitis group, 0.30 for the Japanese HCC group, and 0.28 for the Italian HCC group. The networks were visualized using the Cytoscape software platform [24].

### Other statistical analysis

Methylation levels of DMRs between two groups were evaluated using Hotelling’s *T*-squared test of the Hotelling R-package (version 1.0-5).

## Results

### NAFLD-related networks in viral hepatitis and HCC

**Fig. 1:**
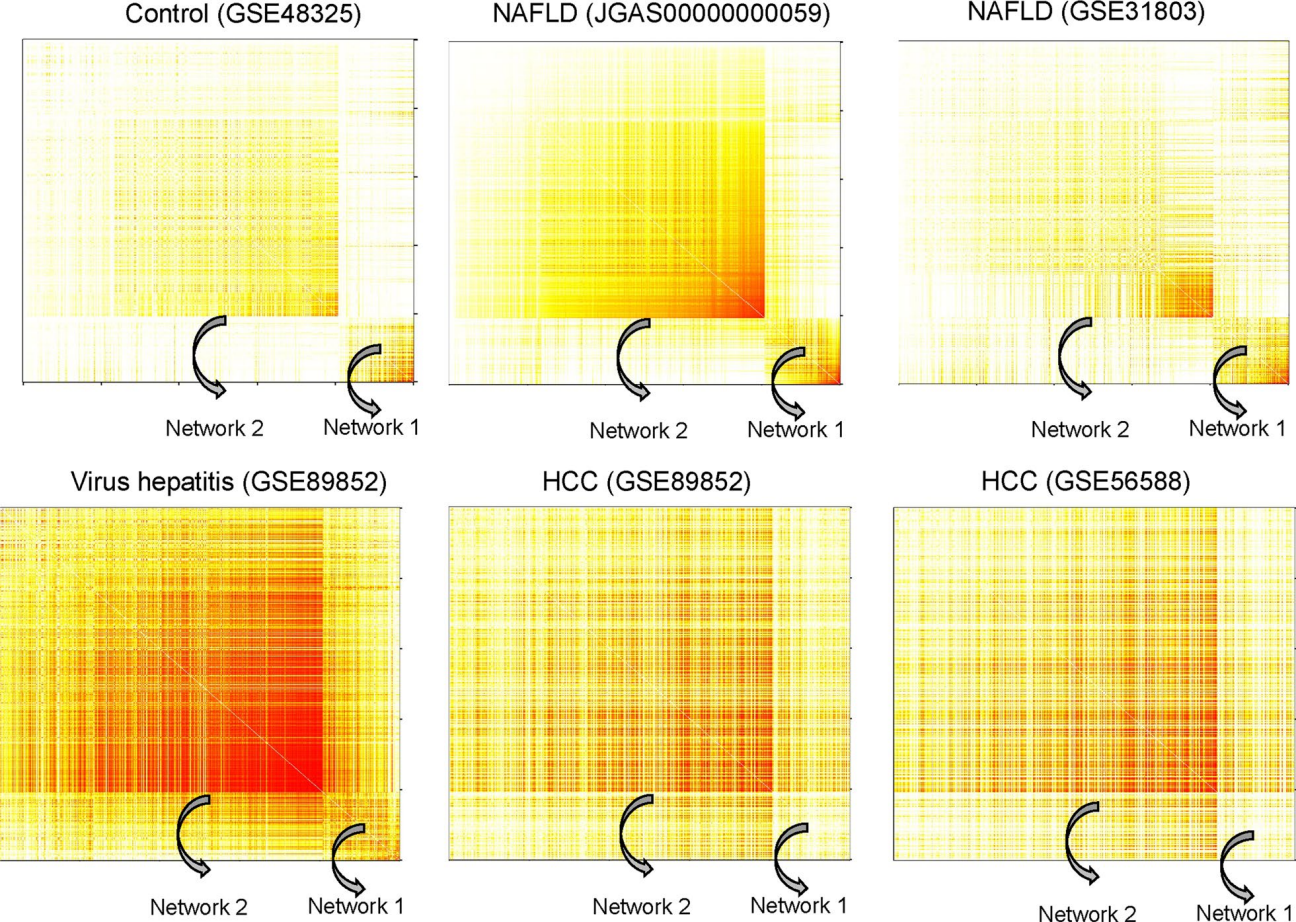
Heatmaps of differentially methylated regions (DMRs) in the control, nonalcoholic fatty liver disease (NAFLD), viral hepatitis, and hepatocellular carcinoma (HCC) datasets. Average values of topological overlap measures for the CpG matrix combining two different DMRs were calculated as described in the “Methods” section. Heatmaps of 610 DMRs were constructed using the German control, Japanese NAFLD, American NAFLD, Japanese viral hepatitis, Japanese HCC, and Italian HCC datasets. The order of the DMRs was the same in each of the six heatmaps. Light colors represent low topological overlap with the progressively darker red color indicating increasing overlap

We have previously reported two DMR networks associated with the progression of NAFLD [14], as shown in Fig. 1. DMR network 1 was observed in the control and the two NAFLD populations. However, this network was not observed in the non-cancerous viral hepatitis or the two HCC populations, suggesting this DMR network was specific to normal and NAFLD conditions. Meanwhile, DMR network 2 was not obvious in the normal group, but was observed in the two NAFLD populations and in the non-cancerous viral hepatitis and two HCC populations.

**Fig. 2:**
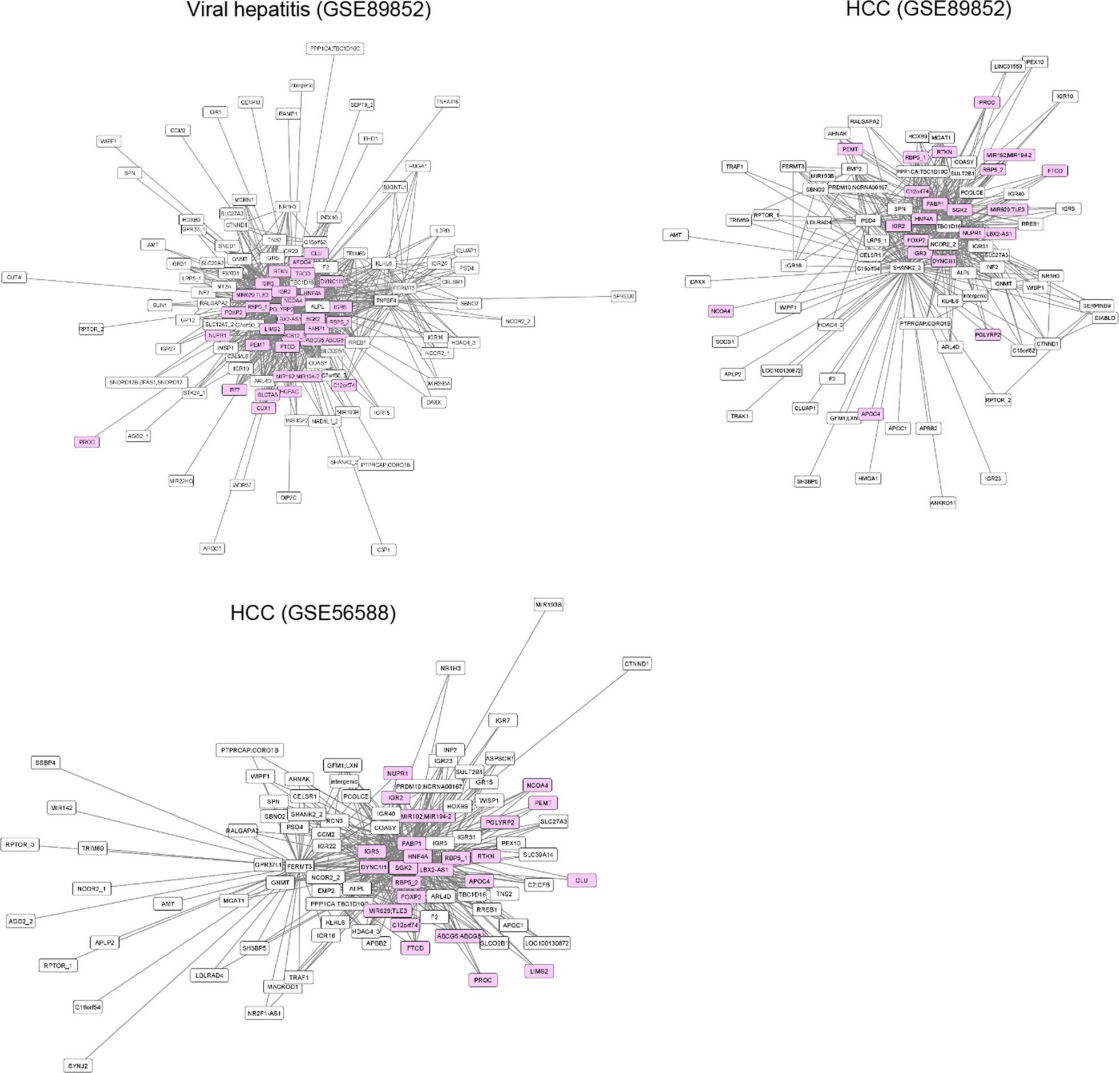
Differentially methylated region (DMR) network 2 in the Japanese viral hepatitis, Japanese hepatocellular carcinoma (HCC), and Italian HCC datasets. The DMR network 2 of Japanese viral hepatitis, Japanese HCC, and Italian HCC were extracted as described in the “Methods” section. Nodes (DMRs) identified in the Japanese and American nonalcoholic fatty liver disease **(**NAFLD) groups in our previous study [14], are indicated in pink

After visualization using the Cytoscape platform, it was determined that all the genes of the DMR network 2 in NAFLD were present in the DMR network observed in viral hepatitis (Fig. 2). Moreover, many of the genes of DMR network 2 were also observed in HCC (Fig. 2). The top 10 genes (nodes) connected with many other genes in the two HCC populations are shown in Table 1. Fatty acid binding protein 1 (*FABP1*), serum/glucocorticoid regulated kinase 2 (*SGK2*), and hepatocyte nuclear factor 4 α (*HNF4A*) were identified as potential hub genes of DMR network 2 in HCC. Detailed information regarding the CpG sites of DMR network 2, which were commonly observed in NAFLD and HCC populations, is provided in Additional file 1: Table S1.

**Table 1:** Top 10 nodes (genes) connected with many other nodes observed in networks of hepatocellular carcinoma (HCC)

GSE89852		GSE56588	
Node (gene)	No. of edges (%)	Node (gene)	No. of edges (%)
SHANK2	81 (9.8)	FABP1	82 (9.9)
SGK2	54 (6.6)	SGK2	79 (9.5)
FABP1	51 (6.2)	FERMT3	61 (7.3)
TBC1D16	50 (6.1)	DYNC1I1	54 (6.5)
PSD4	33 (4.0)	LBX2-AS1	53 (6.4)
NCOR2	31 (3.8)	HNF4A	44 (5.3)
HNF4A	28 (3.4)	RBP5_2	39 (4.7)
FOXP2	27 (3.3)	ALPL	25 (3.0)
IGR2	27 (3.3)	C12orf74	12 (1.4)
IGR3	27 (3.3)	MIR192;MIR194-2	10 (1.2)
Total	824 (100)		832 (100)

*IGR* intergenic region

### Methylation levels of DMR network 2 genes in HCC

**Fig. 3:**
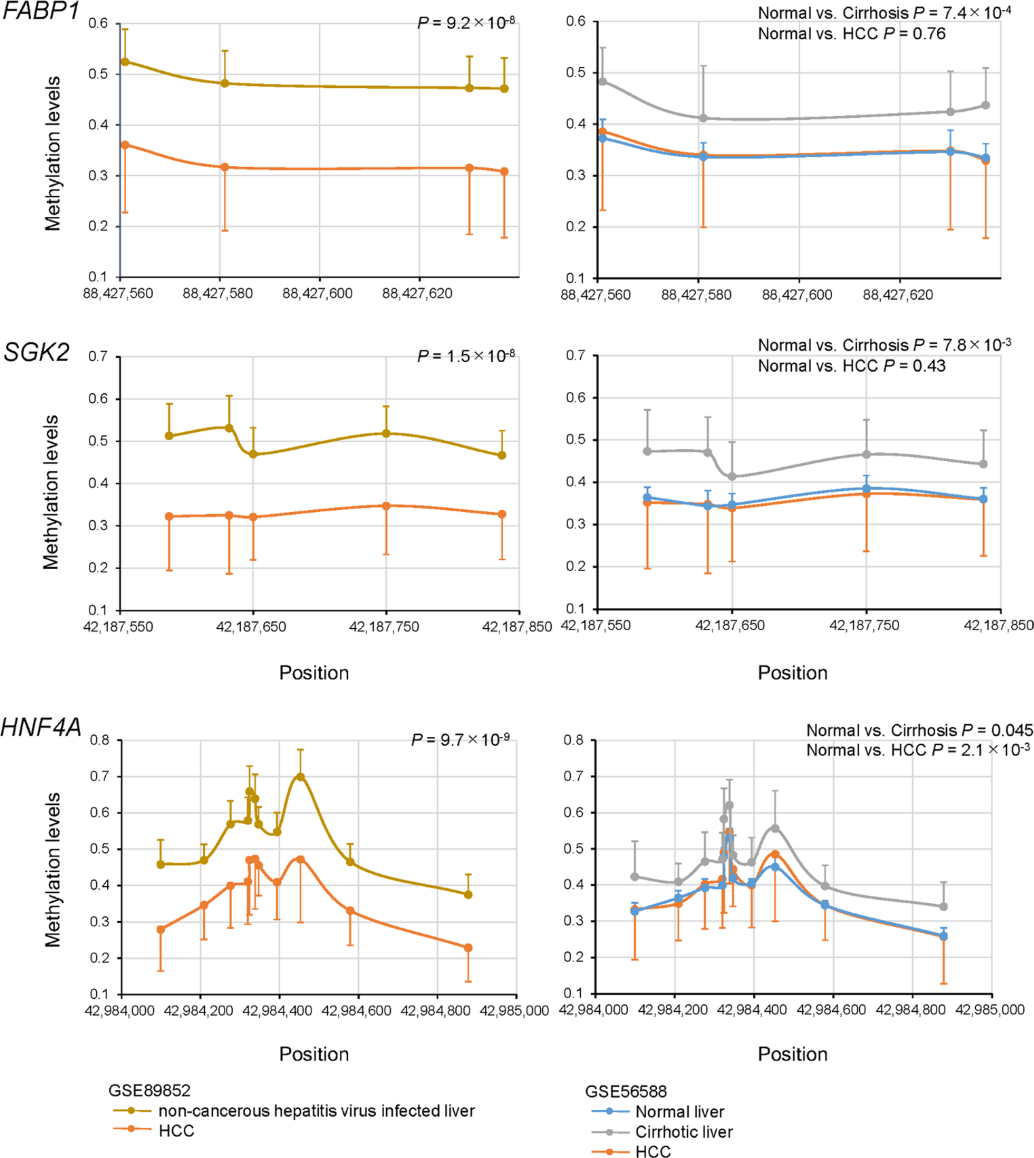
Differentially methylated regions (DMRs) of genes, *FABP1*, *SGK2*, and *HNF4A* in livers of the patients with hepatocellular carcinoma (HCC). Data are expressed as mean ± standard deviation. The left panels show a comparison between paired liver samples from viral hepatitis and HCC. The right panels show a comparison of liver samples from normal, cirrhosis and HCC groups. *P* values were calculated using Hotelling’s *T*-squared test

Next, we examined the methylation levels of *FABP1*, *SGK2*, and *HNF4A* (Fig. 3). The levels of methylation of these genes in HCC (GSE89852) were significantly decreased compared to those in non-cancerous paired samples of hepatitis virus-infected liver (Fig. 3). The GSE56588 dataset includes methylation levels for 11 normal liver samples and 10 cirrhotic liver samples. Although the sample sizes of normal and cirrhotic livers were small, the levels of *FABP1*, *SGK2*, and *HNF4A* methylation in cirrhotic livers were higher than those in normal livers. Methylation levels for HCC samples were comparable to normal levels. The methylation levels of genes in DMR network 2 correlated with increased fibrosis stages in NAFLD [13, 14]. Therefore, the increased methylation levels of these genes in viral hepatitis and cirrhotic liver could also be related to increased fibrosis. Methylation levels of the other 18 genes of the DMR network 2 were higher in non-cancerous hepatitis virus-infected liver samples compared to those in HCC samples (Additional file 2: Fig. S1, GSE89852). Evaluation of the GSE56588 dataset revealed the methylation levels of these 18 genes in cirrhotic livers were higher than those in normal livers. Methylation levels of these genes were decreased in HCC tissues.

### Methylation levels of FABP1, SGK2, and HNF4A in other cirrhosis and HCC liver samples as well as HCC cell lines

**Fig. 4:**
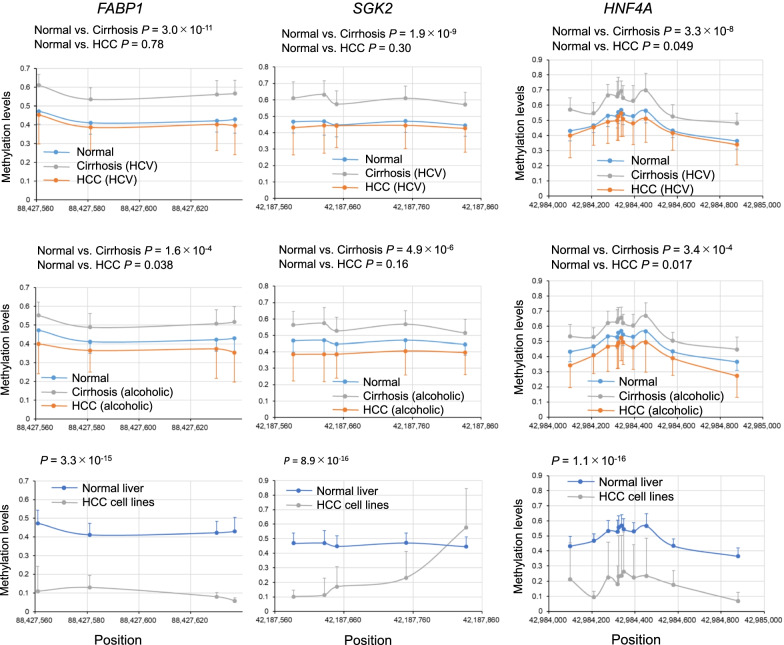
Differentially methylated regions (DMRs) of genes, *FABP1*, *SGK2*, and *HNF4A* in cirrhotic livers, and livers from patients with hepatocellular carcinoma (HCC), and HCC cell lines. Data are expressed as mean ± standard deviation. Data were analyzed using the GSE60753 dataset. The upper panels show a comparison of liver samples from control patients, patients with HCV-infected cirrhosis, and patients with HCC. The middle panels show a comparison of liver samples from control patients, patients with alcoholic cirrhosis, and patients with HCC arising from chronic alcoholism. The lower panels show a comparison between normal liver tissues and HCC cell lines. *P* values were calculated using Hotelling’s *T*-squared test

To evaluate whether *FABP1*, *SGK2*, and *HNF4A* related to cirrhosis and HCC, we examined the methylation levels of these gene using the GSE60753 dataset. The GSE60753 dataset includes methylation levels for 34 normal liver samples, 39 hepatitis C virus (HCV)-infected and 21 alcoholic cirrhotic liver samples, and 12 HCV-infected and 15 alcohol-related HCC samples [19]. As observed in the GSE56588 dataset, the methylation levels of *FABP1*, *SGK2*, and *HNF4A* in cirrhotic livers were higher than those in normal livers. The higher methylation levels were observed in cirrhosis of different causes, such as HCV virus and alcohol (Fig. 4). Methylation levels for HCC samples were comparable to normal levels, even if the causes of HCC were different. These data indicated the dynamic change of the methylation levels of *FABP1*, *SGK2*, and *HNF4A*, which were increased during fibrosis progression and decreased after HCC development.

Using data of eight established HCC cell lines obtained from GSE60753, we found that the methylation levels of *FABP1*, *SGK2*, and *HNF4A* were significantly lower compared to those of normal liver tissues (Fig. 4). The changes in methylation levels of these genes were confirmed at the cell level.

## Discussion

Epigenetic studies are considered to be important for better understanding the pathogenesis of liver fibrosis and HCC. Many studies have been conducted [7, 11, 13–19]; however, the changes in methylation levels during liver disease progression are still controversial. Different from an expression analysis, many CpG sites affecting expression are located in one gene. To evaluate the methylation levels of multiple consecutive CpG sites, a DMR analysis was proposed and its methods have been developed [20, 25, 26]. Gene network analysis is effective to extract key genes related to the development and progression of diseases [21–23]. For applying the network analysis to evaluate methylation levels in NAFLD, we focused on using the average values of topological overlap measures for the CpG matrix in DMR units to extract DMR networks by combining two different DMRs [14]. Our method could evaluate multiple methylation sites on a gene-by-gene basis and found two DMR networks that strongly correlate with the fibrosis stages of NAFLD.

In the current study, we examined whether these DMR networks were present in viral hepatitis and HCC. DMR network 2 was clearly observed in the viral hepatitis and two HCC populations that were evaluated. The levels of CpGs methylation in DMR network 2 were increased in viral hepatitis and cirrhosis, consistent with that in advanced NAFLD [14]. This suggests that DMR network 2 may play important roles in viral hepatitis fibrosis, as well as NAFLD fibrosis. In contrast to methylation levels in advanced NAFLD, viral hepatitis, and cirrhosis, the methylation levels in HCC were decreased, even though DMR network 2 was conserved in HCC. These findings suggest that DMR network 2 plays an important role in carcinogenesis in advanced NAFLD and viral hepatitis.

The main genes involved in DMR network 2 were *FABP1*, *SGK2*, and *HNF4A*. The methylation levels of these gene were increased in liver fibrosis due to HCV infection or chronic alcoholism as well as NAFLD, and were decreased in HCC with HCV infection or alcoholism. FABP1 is primarily expressed in the liver, where it is involved in the binding, transport, and metabolism of long-chain fatty acids. Furthermore, FABP1 plays important roles in changes of cellular lipid metabolic homeostasis that are associated with liver diseases, such as NAFLD, viral hepatitis, cirrhosis, and HCC. FABP1 may also play a protective role in hepatocytes by controlling the availability of long-chain fatty acids and their oxidative metabolites, which are potentially cytotoxic [27]. Therefore, increased methylation levels of *FABP1* could deteriorate the protection of hepatocytes from oxidative metabolites in NAFLD as well as in viral and alcoholic hepatitis, while decreased *FABP1* methylation levels in HCC may be related to the promotion of cell growth.

SGK2 is an isoform of the serum and glucocorticoid kinase (SGK) family of serine/threonine kinases [28]. Expression of *SGK2* is induced in response to signals that activate phosphatidylinositol 3-kinase and is implicated in the regulation of cell growth, proliferation, survival, and migration. Thus, increased methylation of *SGK2* may suppress abnormal cell growth in NAFLD as well as in viral and alcoholic hepatitis, while decreased methylation may facilitate cell growth and proliferation in HCC. Baldwin et al. reported that the loss of p53 or SGK2 alone has little effect on cell viability, whereas loss of both p53 and SGK2 leads to cell death, indicating synthetic lethality [29]. Accordingly, SGK2 could theoretically be a target for tumor suppressor-specific drug discovery.

HNF4A is a transcription factor with important roles in liver development, hepatocyte differentiation, and lipid and glucose metabolism [30]. The expression of *HNF4A* is decreased in NAFLD [13, 14], as well as in viral hepatitis and cirrhosis [30]. The increased methylation of *HNF4A* in viral and alcoholic cirrhosis could be related to the metabolic dysfunction in these diseases. HNF4A also has oncogenic roles. For instance, HNF4A induces multidrug‑resistance via cell apoptosis, promotes oncogenic cellular metabolism, and reduces the levels of reactive oxygen species (ROS) to enhance genomic alterations and promote cell proliferation, invasion, metastasis, and angiogenesis [31]. Decreased methylation levels of *HNF4A* in HCC were similar to those of normal levels, which may be related to carcinogenesis in cases of advanced hepatitis, viral hepatitis, and chronic alcoholism. The overall findings indicate that the formation of DMR networks involving *FABP1*, *SGK2*, and *HNF4A* could cooperatively and/or synergistically affect fibrosis and carcinogenesis in advanced NAFLD and viral and alcoholic hepatitis.

In contrast to DMR network 2, DMR network 1 was observed in normal and NAFLD livers, but not observed in viral hepatitis and HCC. The methylation levels of CpGs in DMR network 1 are strongly correlated with patient age and blood glucose levels, in addition to the stages of fibrosis [14]. Patients with NAFLD are often affected by obesity and type 2 diabetes. Thus, long-term hyperglycemia induced by excess calorie intake was considered to promote DMR network 1 in NAFLD. HCV is often accompanied by metabolic alterations, such as dyslipidemia, hepatic steatosis, insulin resistance, obesity, and type 2 diabetes [32]; however, DMR network 1 was not observed in viral hepatitis or in HCC developed from viral hepatitis. HCC develops in the presence of cirrhosis for most cases with viral etiologies, while patients with NAFLD can develop HCC in the absence of cirrhosis [5]. Therefore, DMR network 1 could be related to the carcinogenesis in NAFLD without cirrhosis. This is consistent with the DMR network 1 including genes involved in transcriptional regulation, such as zinc finger and BTB domain containing 38 (*ZBTB38*), transducing-like enhancer of split 3 (*TLE3*), and castor zinc finger 1 (*CASZ1*), and the oncogene agrin (*AGRN)*, which enhances cellular proliferation, migration, and oncogenic signaling [14, 33–36]. Accordingly, these genes in DMR network 1 may be potential targets for the protection from NAFLD progression to HCC.

## Conclusions

By performing DMR network analyses, we identified the NAFLD-specific DMR network 1 and DMR network 2, which was present in NAFLD, viral hepatitis, and HCC. We also determined that the methylation levels of DMR network 2 changed cooperatively and/or synergistically during the development of HCC. Common changes in the methylation levels of *FABP1*, *SGK2*, and *HNF4A* from fibrosis to cancer were found in NAFLD, hepatitis virus, and alcoholic hepatitis. Our findings provide new insights into the pathogenesis of HCC, with *FABP1*, *SGK2*, and *HNF4A* being candidate targets for the prevention and treatment of HCC.

## Supplementary Information

**Additional file 1.** **Table S1**. Commonly observed genes in HCC (GSE89852 and GSE56588) and NAFLD (JGAS00000000059 and GSE31803).

**Additional file 2.** **Fig. S1**. DMRs of the genes in network 2 in livers of the Japanese and Italian HCC patients.

## Data Availability

The data that support the findings of this study are available from DDBJ (http://trace.ddbj.nig.ac.jp/jga/index.html, accession number JGAS000059), and NCBI Gene Expression Omnibus (accession number GSE31803, GSE89852, GSE56588, GSE48325, and GSE60753).

## Abbreviations

AGRN: agrin
CASZ1: Castor zinc finger 1
DMR: Differentially methylated region
F0–F2: fibrosis stages 0 - 2
F3–F4: fibrosis stages 3 or 4
FABP1: Fatty acid binding protein 1
HCC: Hepatocellular carcinoma
HCV: Hepatitis C virus
HNF4A: Hepatocyte nuclear factor 4 α
NAFLD: Nonalcoholic fatty liver disease
ROS: Reactive oxygen species
SGK2: Serum/glucocorticoid regulated kinase 2
TLE3: Transducing-like enhancer of split 3
WGCNA: Weighted gene co-expression network analysis
ZBTB38: BTB domain containing 38
