# Supplementary material for: Two differentially methylated region networks in nonalcoholic fatty liver disease, viral hepatitis, and hepatocellular carcinoma
Source: BMC Gastroenterol. 2022 Jun 2;22:278. doi: 10.1186/s12876-022-02360-4 (PMC9164838; doi:10.1186/s12876-022-02360-4)
Supplement: Supplementary file 1 — Additional file 1. Table S1. Commonly observed genes in HCC (GSE89852 and GSE56588) and NAFLD (JGAS00000000059 and GSE31803). [file 12876_2022_2360_MOESM1_ESM.docx]

Table S1 Commonly observed genes in HCC (GSE89852 and GSE56588) and NAFLD (JGAS00000000059 and GSE31803)

| DMR | Probe | Chr | Position  (hg19) | Annotated  genes | CpG feature | |
| --- | --- | --- | --- | --- | --- | --- |
| (node) |  |  |  |  | Location | Island |
| PEMT | cg21605540 | 17 | 17,485,823 | *PEMT* | Body | open sea |
|  | cg02094018 | 17 | 17,485,934 | *PEMT* | Body | open sea |
|  | cg02295973 | 17 | 17,486,105 | *PEMT* | Body | open sea |
| LBX2-AS1 | cg02100410 | 2 | 74,731,354 | *LBX2-AS1* | Body | shore |
|  | cg25251459 | 2 | 74,731,371 | *LBX2-AS1* | Body | shore |
|  | cg13407169 | 2 | 74,731,413 | *LBX2-AS1* | Body | shore |
| RBP5_2 | cg14672128 | 12 | 7,280,912 | *RBP5* | Body | open sea |
|  | cg12074585 | 12 | 7,280,958 | *RBP5* | Body | open sea |
|  | cg20315995 | 12 | 7,280,971 | *RBP5* | Body | open sea |
|  | cg24441911 | 12 | 7,280,988 | *RBP5* | Body | open sea |
|  | cg24319651 | 12 | 7,281,343 | *RBP5* | 1stExon | open sea |
| FTCD | cg04413147 | 21 | 47,575,134 | *FTCD* | Body | open sea |
|  | cg10394047 | 21 | 47,575,416 | *FTCD* | 1stExon | open sea |
|  | cg09436823 | 21 | 47,575,498 | *FTCD* | TSS200 | open sea |
|  | cg18024037 | 21 | 47,575,504 | *FTCD* | TSS200 | open sea |
|  | cg25322086 | 21 | 47,575,547 | *FTCD* | TSS200 | open sea |
| RBP5_1 | cg00294025 | 12 | 7,276,360 | *RBP5* | 3'UTR | open sea |
|  | cg10993460 | 12 | 7,276,482 | *RBP5* | 3'UTR | open sea |
|  | cg16959747 | 12 | 7,276,714 | *RBP5* | 3'UTR | open sea |
| APOC4 | cg17769836 | 19 | 45,445,437 | *APOC4* | TSS200 | open sea |
|  | cg04401876 | 19 | 45,445,449 | *APOC4* | TSS200 | open sea |
|  | cg04347059 | 19 | 45,445,486 | *APOC4* | TSS200 | open sea |
|  | cg02912790 | 19 | 45,445,491 | *APOC4* | TSS200 | open sea |
|  | cg27353824 | 19 | 45,445,521 | *APOC4* | 5'UTR | open sea |
|  | cg25017250 | 19 | 45,445,693 | *APOC4* | Body | open sea |
| PGLYRP2 | cg17915429 | 19 | 15,590,069 | *PGLYRP2* | Body | open sea |
|  | cg17752089 | 19 | 15,590,308 | *PGLYRP2* | 5'UTR | open sea |
|  | cg09054960 | 19 | 15,590,328 | *PGLYRP2* | TSS200 | open sea |
|  | cg22310770 | 19 | 15,590,368 | *PGLYRP2* | TSS200 | open sea |
|  | cg07408456 | 19 | 15,590,532 | *PGLYRP2* | TSS1500 | open sea |
|  | cg17473673 | 19 | 15,590,570 | *PGLYRP2* | TSS1500 | open sea |
| HNF4A | cg08314996 | 20 | 42,984,099 | *HNF4A* | TSS1500 | open sea |
|  | cg16121136 | 20 | 42,984,209 | *HNF4A* | TSS1500 | open sea |
|  | cg24084358 | 20 | 42,984,276 | *HNF4A* | TSS200 | open sea |
|  | cg06126829 | 20 | 42,984,320 | *HNF4A* | TSS200 | open sea |
|  | cg06640637 | 20 | 42,984,324 | *HNF4A* | TSS200 | open sea |
|  | cg20848979 | 20 | 42,984,338 | *HNF4A* | TSS200 | open sea |
|  | cg22958104 | 20 | 42,984,347 | *HNF4A* | TSS200 | open sea |
|  | cg16221969 | 20 | 42,984,394 | *HNF4A* | TSS200 | open sea |
|  | cg23792485 | 20 | 42,984,453 | *HNF4A* | 1stExon | open sea |
|  | cg21081369 | 20 | 42,984,579 | *HNF4A* | Body | open sea |
|  | cg19717150 | 20 | 42,984,878 | *HNF4A* | Body | open sea |
| MIR192;MIR194-2 | cg05560951 | 11 | 64,658,226 | *MIR192;MIR194-2* | IGR | open sea |
|  | cg02258444 | 11 | 64,658,622 | *MIR192;MIR194-2* | Body | shelf |
|  | cg27083891 | 11 | 64,658,726 | *MIR192;MIR194-2* | TSS200 | shelf |
|  | cg09349409 | 11 | 64,658,765 | *MIR192;MIR194-2* | TSS200 | shelf |
|  | cg18262830 | 11 | 64,658,819 | *MIR192;MIR194-2* | TSS200 | shelf |
|  | cg24803202 | 11 | 64,658,903 | *MIR192;MIR194-2* | Body | shelf |
|  | cg08432452 | 11 | 64,658,936 | *MIR192;MIR194-2* | TSS1500 | shelf |
|  | cg00589493 | 11 | 64,658,940 | *MIR192;MIR194-2* | TSS1500 | shelf |
|  | cg13092487 | 11 | 64,658,946 | *MIR192;MIR194-2* | TSS1500 | shelf |
|  | cg24154336 | 11 | 64,659,044 | *MIR192;MIR194-2* | TSS1500 | shelf |
|  | cg00400165 | 11 | 64,659,060 | *MIR192;MIR194-2* | TSS1500 | shelf |
|  | cg00376448 | 11 | 64,659,065 | *MIR192;MIR194-2* | TSS1500 | shelf |
|  | cg02494703 | 11 | 64,659,387 | *MIR192;MIR194-2* | TSS1500 | shelf |
| MIR629;TLE3 | cg05185738 | 15 | 70,371,992 | *MIR629;TLE3* | TSS200 | open sea |
|  | cg17972789 | 15 | 70,372,105 | *MIR629;TLE3* | Body | open sea |
|  | cg13912196 | 15 | 70,372,127 | *MIR629;TLE3* | Body | open sea |
|  | cg02852421 | 15 | 70,372,614 | *MIR629;TLE3* | Body | open sea |
| NCOA4 | cg06098215 | 10 | 51,575,702 | *NCOA4* | 5'UTR | shelf |
|  | cg16814786 | 10 | 51,575,763 | *NCOA4* | 5'UTR | shelf |
|  | cg01315067 | 10 | 51,576,232 | *NCOA4* | TSS200 | shelf |
|  | cg00302587 | 10 | 51,576,241 | *NCOA4* | TSS200 | shelf |
|  | cg20166027 | 10 | 51,576,452 | *NCOA4* | 1stExon | shelf |
| LIMS2 | cg13099839 | 2 | 128,422,113 | *LIMS2* | Body | island |
|  | cg16944093 | 2 | 128,422,120 | *LIMS2* | Body | island |
|  | cg10661054 | 2 | 128,422,179 | *LIMS2* | Body | island |
|  | cg19426955 | 2 | 128,422,224 | *LIMS2* | Body | shore |
|  | cg11535366 | 2 | 128,422,284 | *LIMS2* | Body | shore |
|  | cg18044111 | 2 | 128,422,307 | *LIMS2* | Body | shore |
|  | cg21339084 | 2 | 128,422,433 | *LIMS2* | Body | shore |
|  | cg14489933 | 2 | 128,422,619 | *LIMS2* | Body | shore |
|  | cg19326543 | 2 | 128,422,715 | *LIMS2* | Body | shore |
|  | cg15781838 | 2 | 128,422,717 | *LIMS2* | Body | shore |
| FABP1 | cg19910382 | 2 | 88,427,561 | *FABP1* | 1stExon | open sea |
|  | cg19217130 | 2 | 88,427,581 | *FABP1* | TSS200 | open sea |
|  | cg24046616 | 2 | 88,427,630 | *FABP1* | TSS200 | open sea |
|  | cg24933157 | 2 | 88,427,637 | *FABP1* | TSS200 | open sea |
| NUPR1 | cg08818284 | 16 | 28,548,873 | *NUPR1* | 3'UTR | open sea |
|  | cg06723057 | 16 | 28,549,923 | *NUPR1* | Body | open sea |
|  | cg05590982 | 16 | 28,550,171 | *NUPR1* | 1stExon | open sea |
|  | cg04492847 | 16 | 28,550,525 | *NUPR1* | TSS200 | open sea |
|  | cg06288570 | 16 | 28,550,567 | *NUPR1* | TSS200 | open sea |
|  | cg15149645 | 16 | 28,550,619 | *NUPR1* | TSS200 | open sea |
|  | cg01542023 | 16 | 28,550,637 | *NUPR1* | TSS200 | open sea |
| PROC | cg25457027 | 2 | 128,175,179 | *PROC* | TSS1500 | shore |
|  | cg10021288 | 2 | 128,175,891 | *PROC* | TSS200 | shelf |
|  | cg22856114 | 2 | 128,175,905 | *PROC* | TSS200 | shelf |
|  | cg06038358 | 2 | 128,176,007 | *PROC* | 5'UTR | shelf |
|  | cg26718585 | 2 | 128,176,454 | *PROC* | 5'UTR | shelf |
|  | cg11143063 | 2 | 128,177,475 | *PROC* | 5'UTR | shelf |
| IGR2 | cg11847956 | 5 | 173,198,447 | intergenic | IGR | open sea |
|  | cg11212451 | 5 | 173,198,508 | intergenic | IGR | open sea |
|  | cg10818423 | 5 | 173,198,602 | intergenic | IGR | open sea |
| C12orf74 | cg04614923 | 12 | 93,096,622 | *C12orf74* | TSS1500 | open sea |
|  | cg12755471 | 12 | 93,096,780 | *C12orf74* | TSS200 | open sea |
|  | cg27576271 | 12 | 93,096,811 | *C12orf74* | TSS200 | open sea |
|  | cg26383838 | 12 | 93,096,819 | *C12orf74* | TSS200 | open sea |
|  | cg25382573 | 12 | 93,096,844 | *C12orf74* | 5'UTR | open sea |
| RTKN | cg03295928 | 2 | 74,669,349 | *RTKN* | TSS1500 | island |
|  | cg14481208 | 2 | 74,669,375 | *RTKN* | TSS1500 | island |
|  | cg00658082 | 2 | 74,669,380 | *RTKN* | TSS1500 | island |
|  | cg26090072 | 2 | 74,669,387 | *RTKN* | TSS1500 | island |
| FOXP2 | cg01306563 | 7 | 114,055,074 | *FOXP2* | 1stExon | open sea |
|  | cg18546840 | 7 | 114,055,123 | *FOXP2* | 1stExon | open sea |
|  | cg24786986 | 7 | 114,055,133 | *FOXP2* | 1stExon | open sea |
|  | cg18871253 | 7 | 114,055,137 | *FOXP2* | 1stExon | open sea |
|  | cg19655952 | 7 | 114,055,204 | *FOXP2* | 1stExon | open sea |
|  | cg02211646 | 7 | 114,055,210 | *FOXP2* | 1stExon | open sea |
| IGR3 | cg08614082 | 6 | 158,375,684 | intergenic | IGR | open sea |
|  | cg27359374 | 6 | 158,375,774 | intergenic | IGR | open sea |
|  | cg11051752 | 6 | 158,375,814 | intergenic | IGR | open sea |
| DYNC1I1 | cg09136052 | 7 | 95,546,508 | *DYNC1I1* | Body | open sea |
|  | cg18450582 | 7 | 95,546,539 | *DYNC1I1* | Body | open sea |
|  | cg23975973 | 7 | 95,546,556 | *DYNC1I1* | Body | open sea |

Body, gene body; Chr, chromosome; IGR, intergenic region; open sea, isolated CpG sites in the genome; shelf, CpG sites existing in a region 2–4 kb from CpG islands; shore, CpG sites existing up to 2 kb from CpG islands; TSS1500, the sequence region from -200 to -1500 bp upstream of the transcription start site (TSS); TSS200, the region from -200 bp upstream of the TSS; UTR, untranslated region.
