# Supplementary material for: Two differentially methylated region networks in nonalcoholic fatty liver disease, viral hepatitis, and hepatocellular carcinoma
Source: BMC Gastroenterol. 2022 Jun 2;22:278. doi: 10.1186/s12876-022-02360-4 (PMC9164838; doi:10.1186/s12876-022-02360-4)
Supplement: Supplementary file 2 — Additional file 2. Fig. S1. DMRs of the genes in network 2 in livers of the Japanese and Italian HCC patients. [file 12876_2022_2360_MOESM2_ESM.docx]

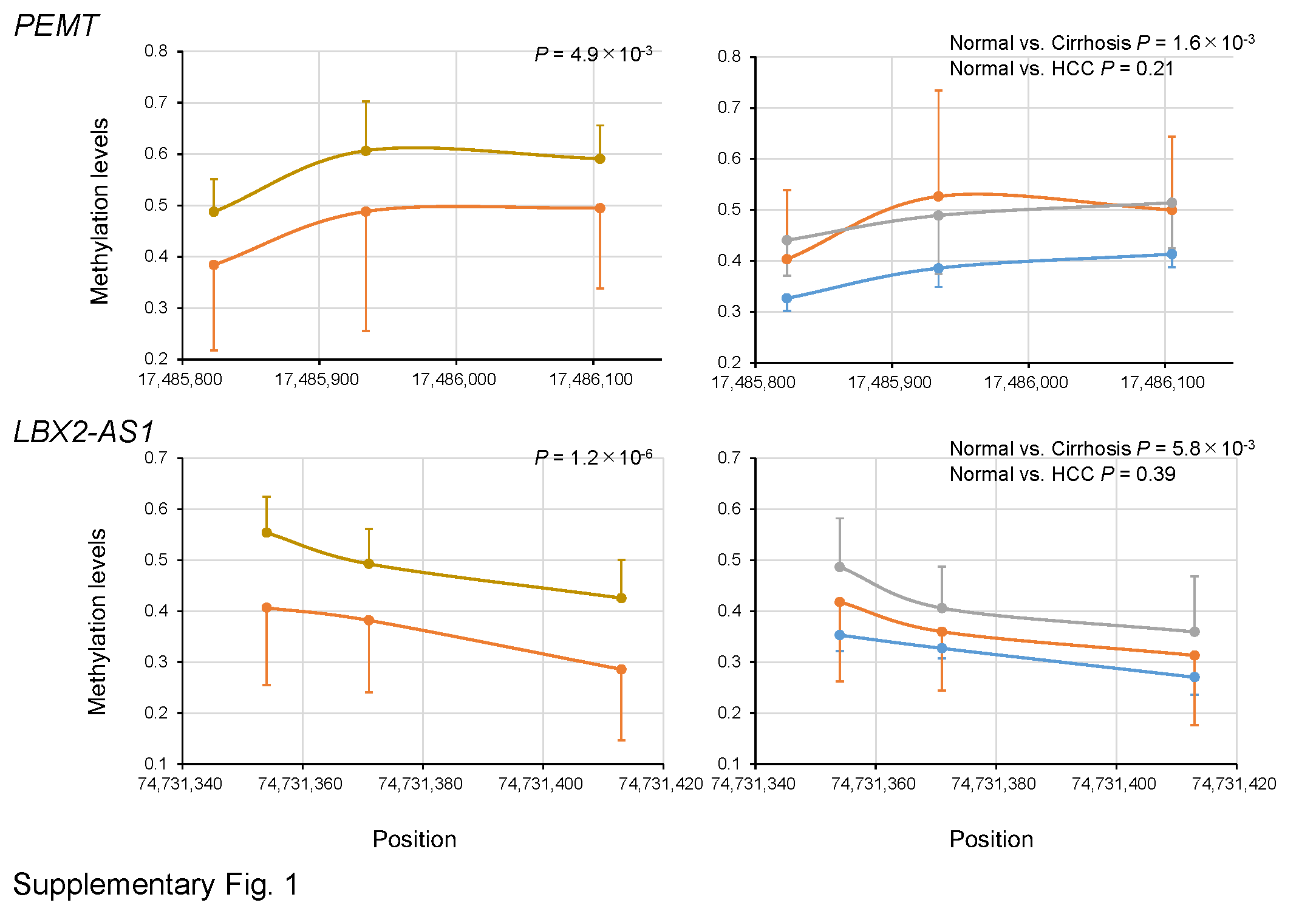


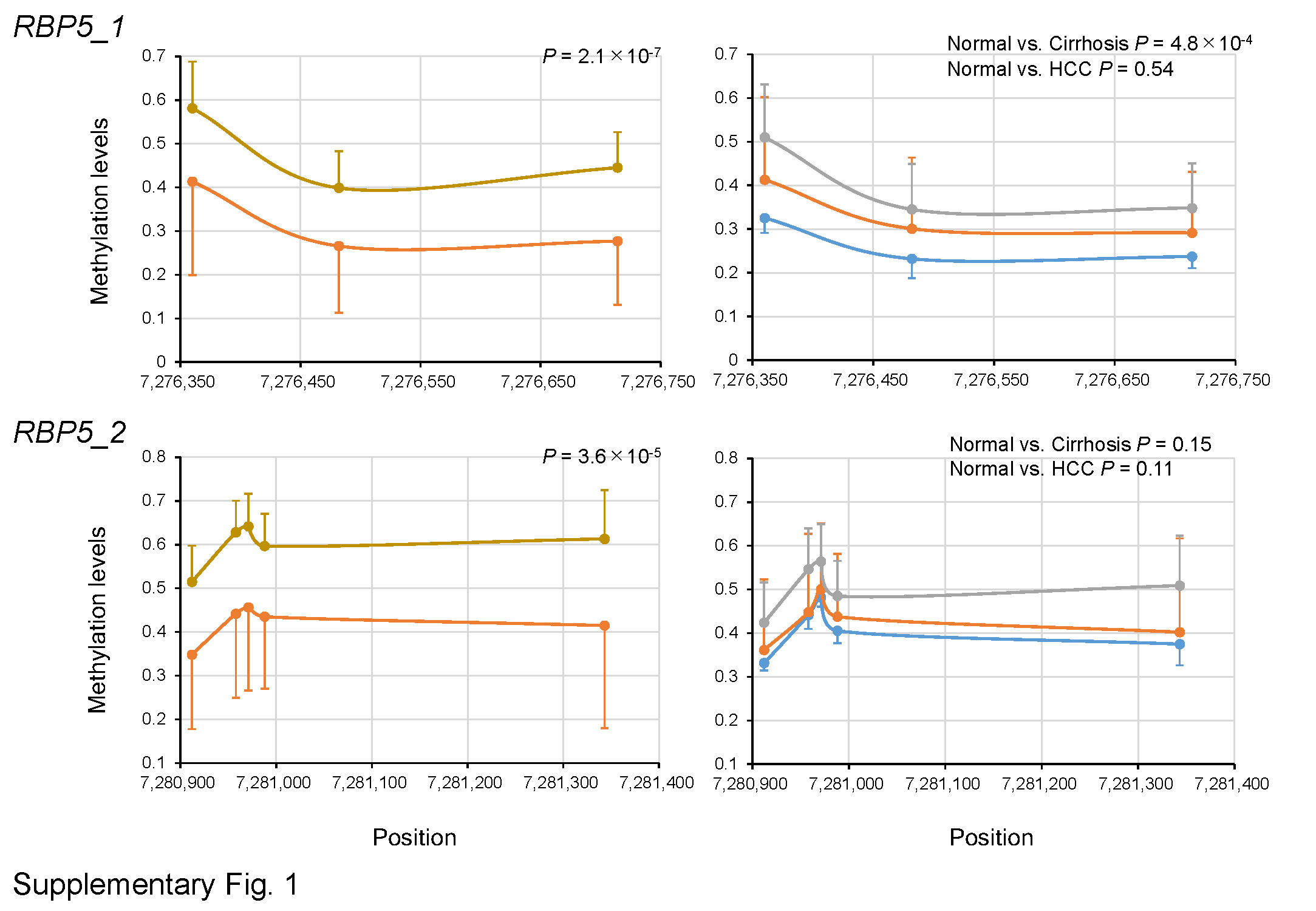


**
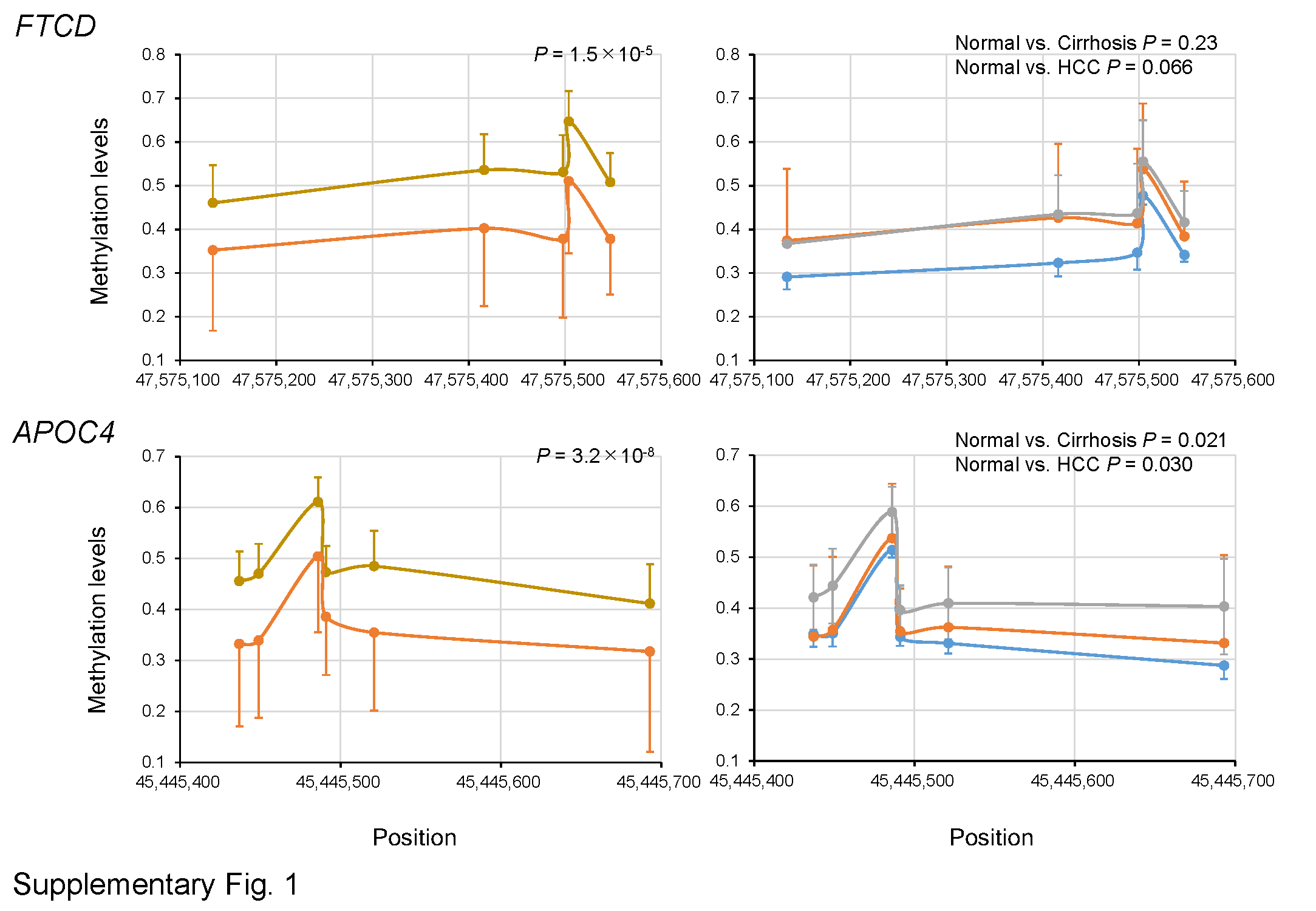
**

**
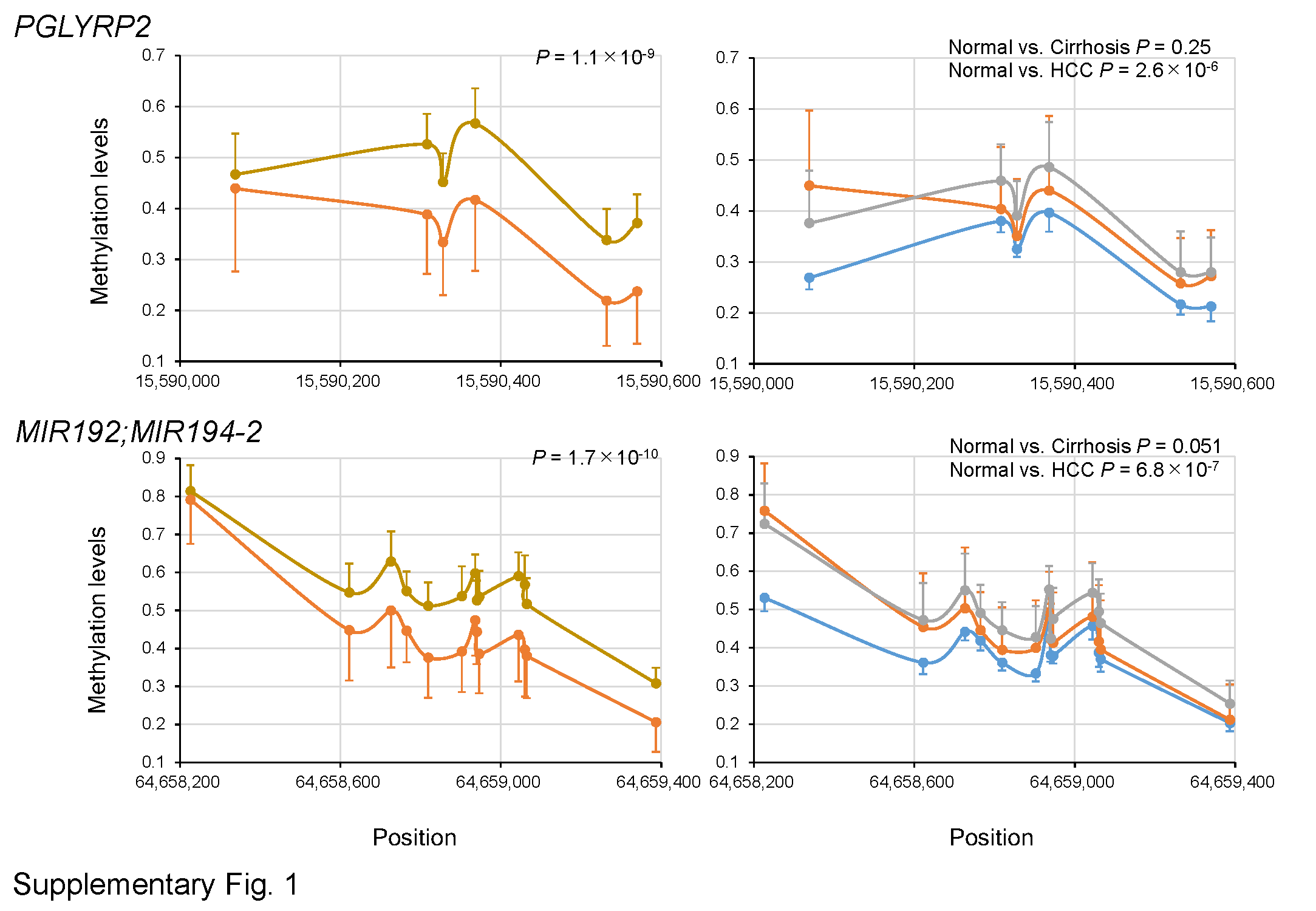
**

**
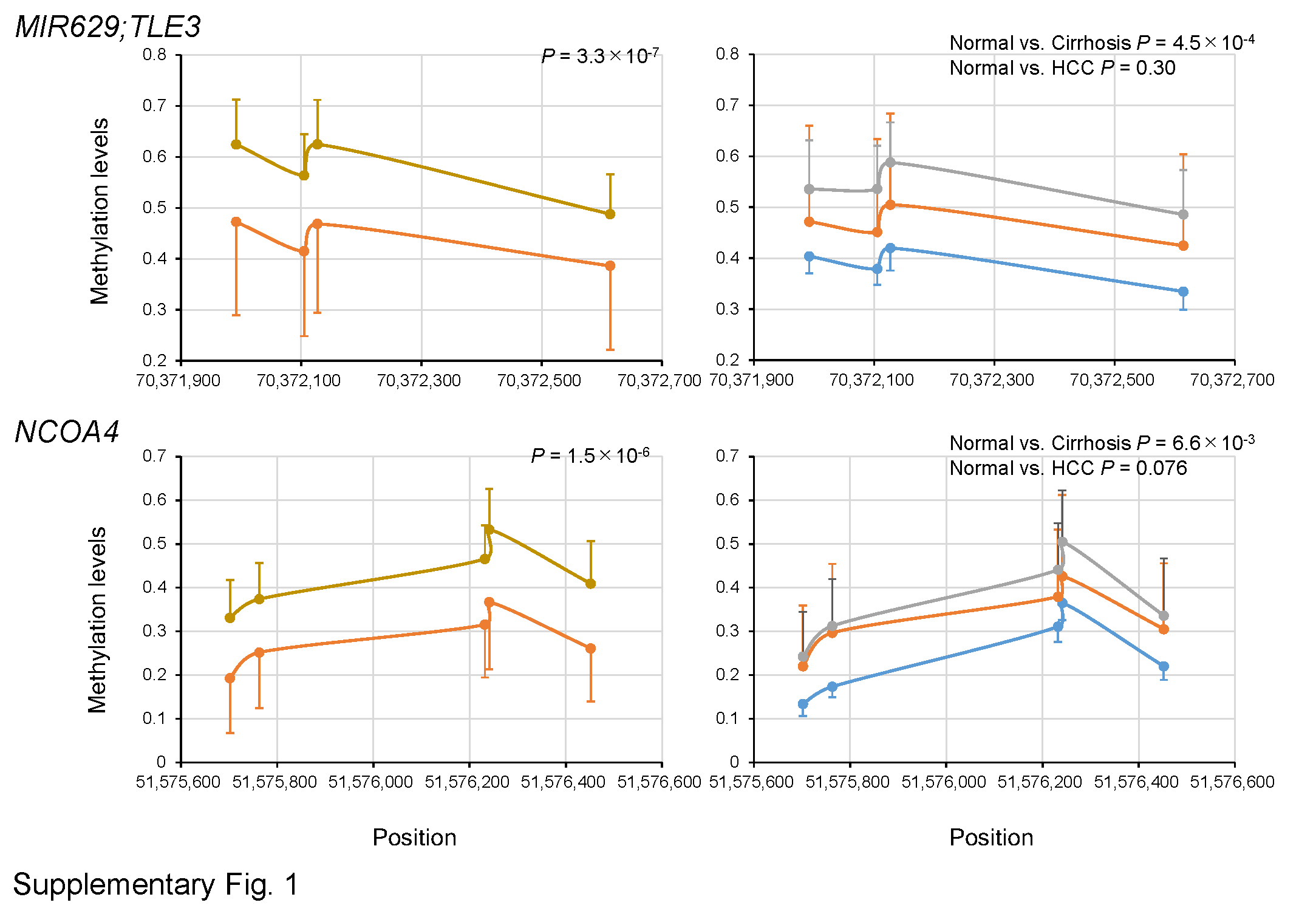

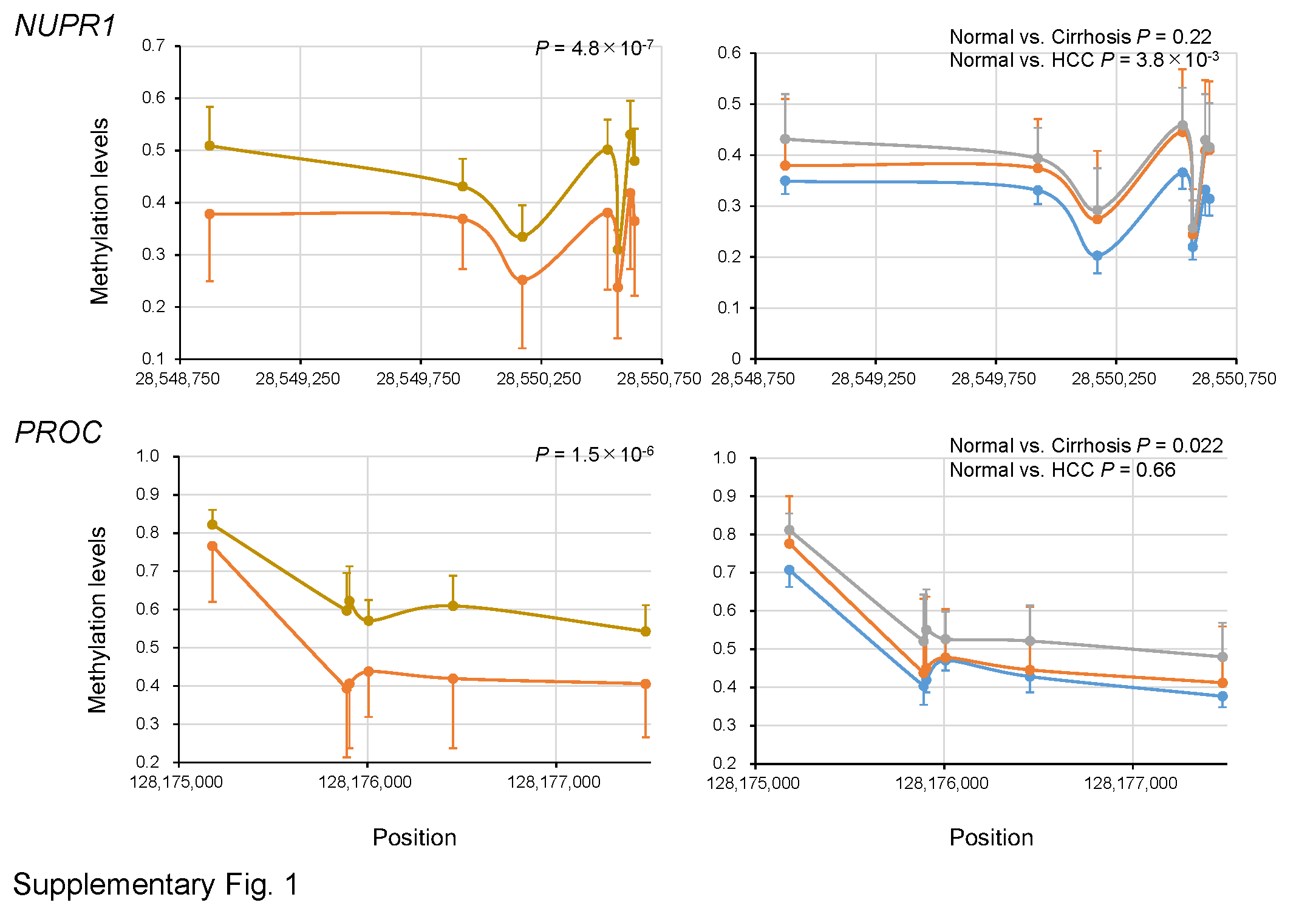

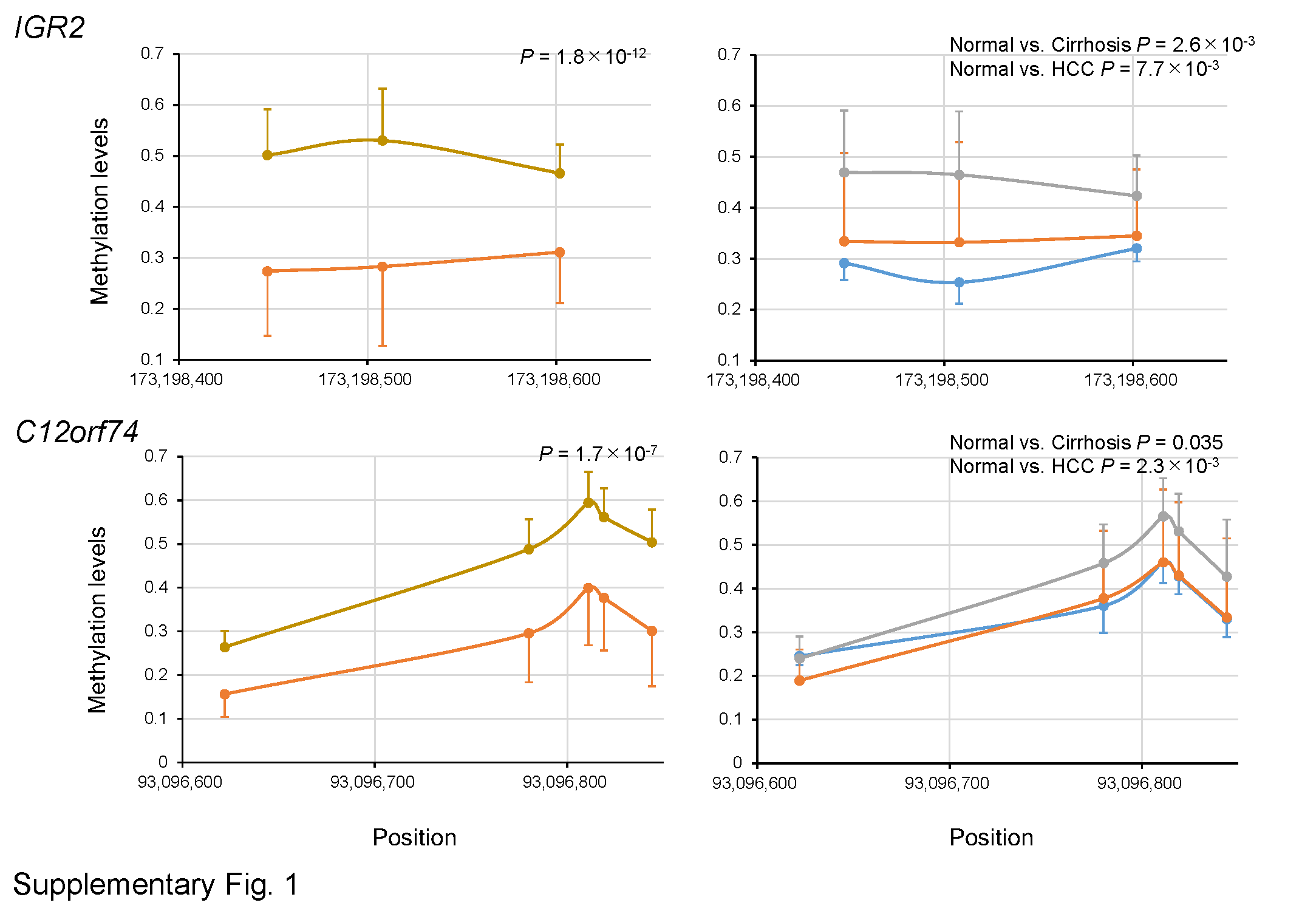

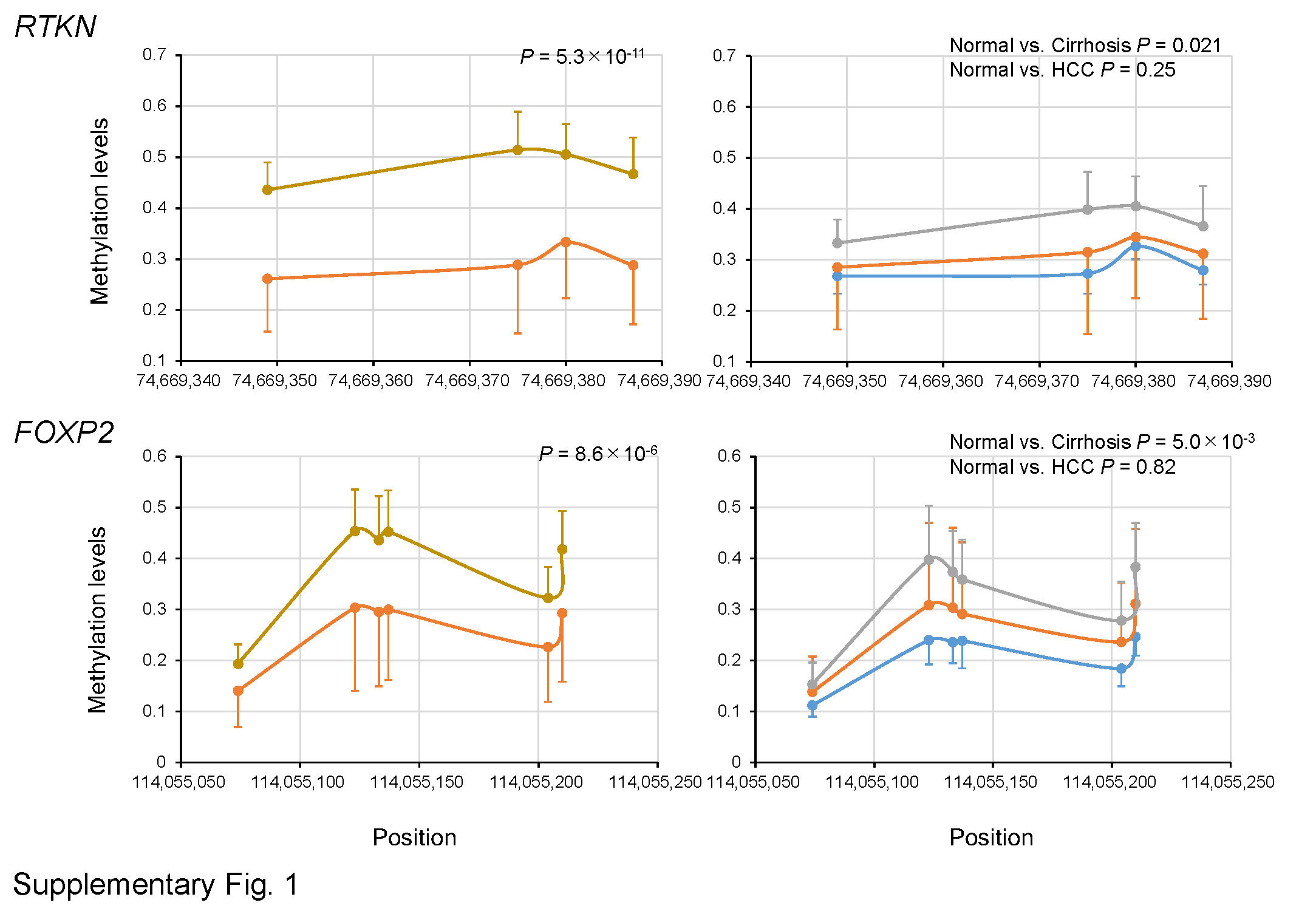

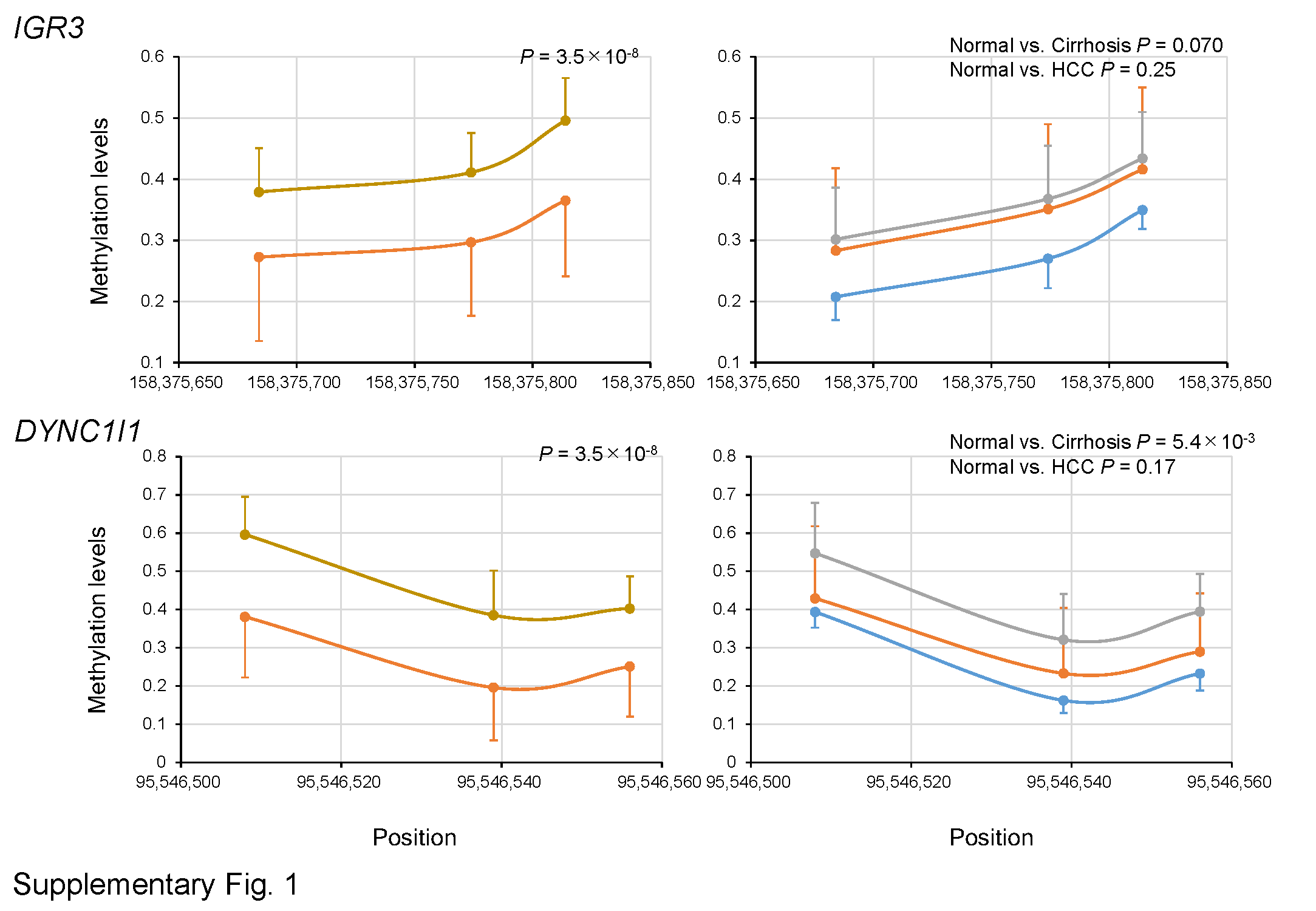
 Fig. S1 DMRs of the genes in network 2 in livers of the Japanese and Italian HCC patients.**

Data are expressed as mean ± standard deviation. The left panels show a comparison between paired liver samples from viral hepatitis and HCC. The right panels show a comparison of liver samples from normal, cirrhosis, and HCC. *P*-values were calculated using Hotelling’s *T*-squared test. IGR, intergenic region.
